# The logic of kinetic regulation in the thioredoxin system

**DOI:** 10.1186/1752-0509-5-15

**Published:** 2011-01-25

**Authors:** Ché S Pillay, Jan-Hendrik S Hofmeyr, Johann M Rohwer

**Affiliations:** 1 Discipline of Genetics, University of KwaZulu-Natal, South Africa, Carbis Road, Pietermaritzburg, 3201, South Africa; 2 Triple-J Group for Molecular Cell Physiology, Department of Biochemistry, Stellenbosch University, Van der Byl Street, Stellenbosch 7600, South Africa; 3 Centre for Studies in Complexity, Stellenbosch University, Marais Street, Stellenbosch, 7600, South Africa

## Abstract

**Background:**

The thioredoxin system consisting of NADP(H), thioredoxin reductase and thioredoxin provides reducing equivalents to a large and diverse array of cellular processes. Despite a great deal of information on the kinetics of individual thioredoxin-dependent reactions, the kinetic regulation of this system as an integrated whole is not known. We address this by using kinetic modeling to identify and describe kinetic behavioral motifs found within the system.

**Results:**

Analysis of a realistic computational model of the *Escherichia coli *thioredoxin system revealed several modes of kinetic regulation in the system. In keeping with published findings, the model showed that thioredoxin-dependent reactions were adaptable (i.e. changes to the thioredoxin system affected the kinetic profiles of these reactions). Further and in contrast to other systems-level descriptions, analysis of the model showed that apparently unrelated thioredoxin oxidation reactions can affect each other via their combined effects on the thioredoxin redox cycle. However, the scale of these effects depended on the kinetics of the individual thioredoxin oxidation reactions with some reactions more sensitive to changes in the thioredoxin cycle and others, such as the Tpx-dependent reduction of hydrogen peroxide, less sensitive to these changes. The coupling of the thioredoxin and Tpx redox cycles also allowed for ultrasensitive changes in the thioredoxin concentration in response to changes in the thioredoxin reductase concentration. We were able to describe the kinetic mechanisms underlying these behaviors precisely with analytical solutions and core models.

**Conclusions:**

Using kinetic modeling we have revealed the logic that underlies the functional organization and kinetic behavior of the thioredoxin system. The thioredoxin redox cycle and associated reactions allows for a system that is adaptable, interconnected and able to display differential sensitivities to changes in this redox cycle. This work provides a theoretical, systems-biological basis for an experimental analysis of the thioredoxin system and its associated reactions.

## Background

**Figure 1:**
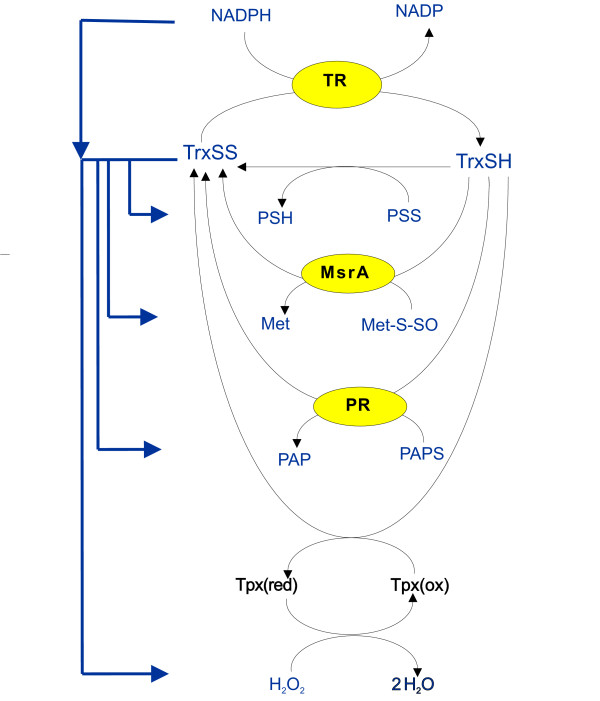
**Modelling the thioredoxin system in *E. coli***. A kinetic model of the thioredoxin system in *E. coli *was developed that included reactions for the reduction of oxidized thioredoxin (TrxSS) by thioredoxin reductase (TR), the thioredoxin-dependent reductions of methionine sulfoxide (Met-S-SO) by methionine sulfoxide reductase (MsrA) and 3'-phosphoadenosine-5'-phosphosulfate (PAPS) by PAPS reductase (PR) and the Tpx-dependent reduction of hydrogen peroxide. In other systems biology approaches, electron flow pathways have been used to model this system (blue arrows). However, in our model, thioredoxin-dependent reactions were modeled as a series of moiety conserved cycles (see text for details).

The thioredoxin redox cycle consisting of NADP(H), thioredoxin reductase and thioredoxin is central to the regulation of several cellular redox processes [1-4]. Thioredoxin reductase reduces the oxidized form of thioredoxin with NADPH as a source of reducing equivalents (Figure 1). Reduced thioredoxin in turn reduces a diverse array of cellular redox partners which are essential in a number of cellular processes such as hydrogen peroxide metabolism, sulfate assimilation, DNA synthesis and signal transduction [1-3,5].

The kinetics of individual thioredoxin-dependent reactions have been studied in great detail; parameters and kinetic models (mass action, ping-pong and redox cycles) are available for many reactions. However, the kinetic regulation of the thioredoxin system as a whole is not known. While kinetic modeling would be the ideal tool to explore this type of regulation, the contrasting *in vivo *and *in vitro *descriptions given to thioredoxins have complicated the construction of models of the thioredoxin system. Redox potentials have been used to describe the thioredoxin system *in vivo *(see for example [6]), which has led to the description of redoxin networks as redox circuits in which thioredoxin is a central node that distributes reducing equivalents to a number of independent processes (Figure 1, [3,7]). On the other hand, thioredoxins have also exhibited enzymatic behaviors such as substrate saturation *in vitro *(see for example [8]), which suggested that Michaelis-Menten parameters were the key descriptors for thioredoxin activity and these parameters have consequently been used to delineate the roles played by individual redoxins in cellular process (see for example [9]). We have recently reconciled these *in vitro *and *in vivo *descriptions by showing that the purported enzymatic properties attributed to thioredoxins resulted from the saturation of the thioredoxin redox cycle and that the ratio of reduced to oxidized thioredoxin reflects the steady state rates of thioredoxin reduction and oxidation [10].

A further challenge for any systems analysis of thioredoxin system is that there is as yet no solid theoretical framework on which to be base such an analysis. It is not clear, for example, whether thioredoxin-dependent reactions affect each other, or how the kinetic structures within the thioredoxin system contribute to the regulation of the system. In this paper we address this by analyzing a set of kinetically distinct reactions from the thioredoxin system in *Escherichia coli *(Figure 1). Using kinetic modeling we precisely describe how changes in the thioredoxin redox cycle affect thioredoxin-dependent reactions and show that the kinetic behavioral motifs found within the thioredoxin system and associated reactions allow for several modes of kinetic regulation.

## Results

### Kinetic model of the *Escherichia coli *thioredoxin system

To identify putative kinetic motifs within the thioredoxin system, a realistic computational model of the *E. coli *thioredoxin system was developed. However, the kinetic parameters for many of the reactions involved in the complete thioredoxin redox network were unavailable or may require revision [10] and we therefore only modeled a set of thioredoxin-dependent reactions (Figure 1, Table 1). To simplify the description of the results below, reduced thioredoxin (Trx1) will be referred to as "thioredoxin" and oxidized thioredoxin will be referred to as "oxidized thioredoxin". Trx2 was not included in the model as its intracellular concentration is substantially lower than Trx1 even under oxidative stress conditions [11].

**Table 1:** Kinetic parameters and metabolite concentrations used in a computational model of the *E. coli *thioredoxin system.

	Value	Reference
**Metabolites**	(*μ*M)	
NADPH	137	[61]
NADP	1	-
TrxSH	1	[62]^a^
TrxSS	1	[62]^a^
PSS	4.23	b
PSH	1	-
MetSO	970	b
Met	4.83 × 10^4^	b
PAPS	0.07	[59]^b, c^
SO_3_^2-^	1	-
PAP	1	-
H_2_O_2_	0.02	[27,28]
H_2_O	1	-
**Thioredoxin**		
**reductase**		
[TR]	4.74 *μ*M	[50]
*k*_cat_	22.75 s^-1^	[63]
*K*_nadph_	1.2 *μ*M	[64]
*K*_trxss_	2.8 *μ*M	[64]
**PSS reduction**		
*k*_2_	0.064 *μ*M^-1^s^-1^	[10]
**Methionine sulfoxide**		
**reductase**		
[MsrA]	2.35 *μ*M	b
*k*_cat_	3.7 s^-1^	[12]
*K*_metso_	1900 *μ*M	[12]
*K*_trxsh_	10 *μ*M	[12]
**PAPS reductase**		
[PR]	0.345 *μ*M	b
*k*_cat_	3.5 s^-1^	[14]
*K*_paps_	22.5 *μ*M	[14]
*K*_trxsh_	13.7 *μ*M	[14]
*k*_pr_	0.156 *μ*M^-1^s^-1^	b
**Tpx**		
[Tpx]	4.88 *μ*M	[57]
*k*_H2O2_	44 *μ*M^-1^.s^-1^	[15]
*k*_trxsh_	3000 *μ*M^-1^.s^-1^	[15]

^a ^Total thioredoxin concentration (TrxSH + TrxSS) obtained from [62]

^b^Discussed in Methods "Realistic model of the *E. coli *thioredoxin system"

^c ^Estimated from bakers yeast

A realistic computational model of the *E. coli *thioredoxin system (Figure 1) containing reactions for the reduction of methionine sulfoxide (MetSO) by methionine sulfoxide reductase A (MsrA), the reduction of 3'-phosphoadenosine-5'-phosphosulfate (PAPS) to adenosine-3'-5'-bisphosphate (PAP) by PAPS reductase, the reduction of the H_2_O_2 _by Tpx and thioredoxin, the reduction of cellular protein disulfides (*PSS*) to protein thiols (*PSH*) by thioredoxin and a thioredoxin reductase reaction was developed. Fixed parameters whose values did not affect the model outputs were set to 1 *μ*M.

In this model, the reduction of oxidized thioredoxin by thioredoxin reductase was assigned Michaelis-Menten kinetics and the non-specific reduction of cytosolic protein disulfides (*PSS*) was modeled with mass action kinetics as previously described [10]. Thioredoxin is an electron donor for the reduction of methionine sulfoxide and 3'-phosphoadenosine-5'-phosphosulfate (PAPS) by methionine sulfoxide and PAPS reductase respectively [5] which both utilize a ping-pong kinetic mechanism [12-14]. However, the concentration for PAPS in our model was lower than the assigned concentration of PAPS reductase (Table 1) and this reaction was therefore modeled with mass action kinetics. The model included the reduction of the peroxiredoxin Tpx by thioredoxin and its oxidation by hydrogen peroxide [15].

Peroxiredoxins have generally been regarded as enzymes and their activities have usually been described with Michaelis-Menten parameters (see for example [15]). However, this description of peroxiredoxin activity is undergoing review for a number of reasons. Firstly, intracellular peroxiredoxin concentrations are usually much greater than the ambient intracellular hydrogen peroxide concentration in cells [16]. Secondly, it has been shown that Michaelis-Menten parameters in general [17] and some assay conditions specifically [18] have underestimated the activity of peroxiredoxins. Dalziel kinetic analysis has also shown that the peroxiredoxin reduction and oxidation reactions for *Schistosoma mansoni *peroxiredoxin 1 can essentially be described by two independent mass action reactions [19], and, in a model of hemoglobin oxidation in red cells, the reduction of hydrogen peroxide by peroxiredoxin II could be modeled effectively with mass action kinetics [20]. In our computational model, the individual oxidation and reduction reactions of Tpx were therefore described with mass action kinetics (i.e. the peroxiredoxin redox cycle was modeled explicitly, see Figure 1).

**Figure 2:**
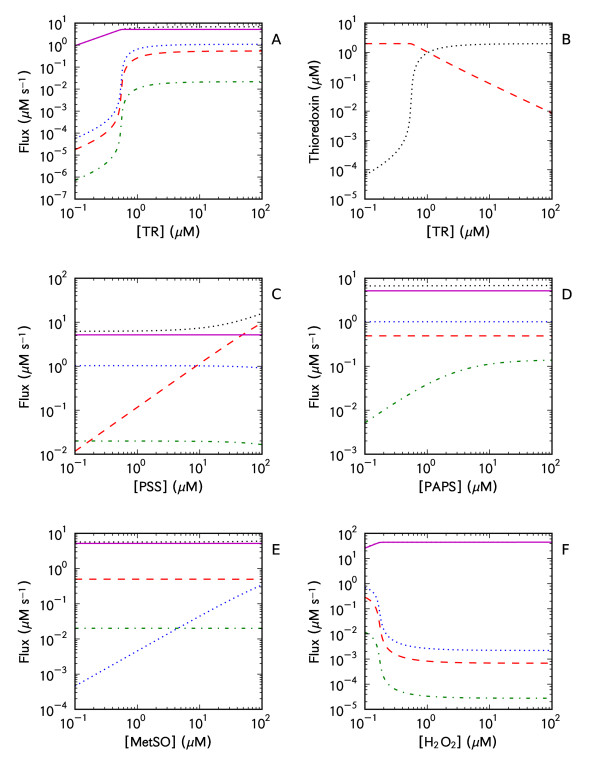
**Parameter portraits of the systemic behavior of a computational model of the *E. coli *thioredoxin system**. Changes in the thioredoxin reductase (TR) concentration affected the fluxes of thioredoxin-dependent reactions (A) and the steady state thioredoxin (blue, •) and oxidized thioredoxin concentrations (red, -) (B) in the model. Similarly, changes in the protein disulfide (C), PAPS (D), methionine sulfoxide (E) and hydrogen peroxide (F) concentrations also affected the fluxes through the system. The PAPS reductase reaction was modeled with mass action kinetics (Figures 2 A-C, E-F) or with ping-pong kinetics (Figure 2D) (see Methods for details). The fluxes shown are thioredoxin reductase (black, •••), protein disulfide reduction (red, ---), methionine sulfoxide reductase (blue, •••), PAPS reductase (green, -•-) and Tpx (magenta, -). Parameters are summarized in Table 1. This figure was generated using 'Logic_Figure 2_and Figure 4.psc' (Additional file 1) and 'Logic_Figure 2 D.psc' (Additional file 2). SBML versions of these models, 'Logic_Figure 2_and Figure 4.xml' (Additional file 5) and 'Logic_Figure 2 D.xml' (Additional file 6), are provided as well.

To analyze this system, model parameters were varied and the effect of these changes on the fluxes through the thioredoxin system was determined [21,22] (Figure 2). Decreases in the concentration of thioredoxin reductase triggered decreases in the fluxes of all thioredoxin-dependent reactions showing that the kinetic profiles for all reactions that yield oxidized thioredoxin can be affected by global changes in the thioredoxin system (Figure 2A). However, the extent of these decreases differed between these reactions with the flux through the Tpx being the least affected and the fluxes for the non-specific reduction of protein disulfides (*PSS*) and the reduction of PAPS being the most affected.

A decrease in the thioredoxin reductase concentration decreased the steady state (reduced) thioredoxin concentration (Figure 2B), consequently affecting the fluxes of the thioredoxin oxidation reactions (Figure 2A). These results agree with published findings which have shown that changes in the thioredoxin system can change the oxidation state of thioredoxin (see for example [23]) and therefore affect thioredoxin-dependent pathways [3,6,23-25]. A striking feature of these changes to the thioredoxin reductase levels was the abrupt, almost ultrasensitive change [26] in the reduced thioredoxin concentration (Figure 2B).

In this model, increases in the protein disulfide concentration (*PSS*) increased the fluxes through the thioredoxin reductase and the protein disulfide reduction reactions (Figure 2C), with the latter reaction saturating at higher concentrations of substrate (not shown) whilst the other reactions were not as affected over the range of concentrations tested in this analysis (Figure 2C). Increases in the substrate concentrations for the PAPS and methionine sulfoxide reductases also increased their fluxes whilst the fluxes of the other thioredoxin oxidation reactions were not dramatically affected (Figure 2D-E). On the other hand, increases in the hydrogen peroxide levels substantially decreased the fluxes through the other thioredoxin oxidation reactions whilst having a smaller effect on the fluxes though the Tpx and the thioredoxin reductase reactions, indicating that the system has a differential sensitivity to hydrogen peroxide (Figure 2F). These results demonstrate that, depending on its kinetics, a given thioredoxin-dependent reaction may be significantly affected by, or may significantly affect other thioredoxin-dependent reactions.

Based on these results we have identified four kinetic behaviors that could potentially regulate the fluxes through the thioredoxin system. Firstly, the system appears to be *adaptable*, i.e. changes in the thioredoxin system can result in different kinetic profiles for thioredoxin-dependent reactions (Figure 2A). Secondly, the (reduced) thioredoxin concentration appears to respond in an *ultrasensitive *manner to changes in the thioredoxin reductase concentration (Figure 2B). Thirdly, reactions involving thioredoxin display varying degrees of *interconnectivity *to each other by virtue of their combined effects on the thioredoxin redox cycle (Figure 2B, C, E). Finally, depending on their kinetics, individual thioredoxin-dependent reactions may be either *insulated *or *sensitive *to changes in the thioredoxin redox cycle (Figure 2C-F). Analytical solutions and computational models were used to investigate the principles underlying these behaviors.

### Adaptable systems

Michaelis-Menten parameters obtained from enzyme-kinetic assays of thioredoxin *in vitro *are not true constants - they have been shown to vary with changes in the concentration of the components of the thioredoxin system (Table 1, [8] and see [10]). Furthermore, changes to the thioredoxin system *in vivo *affect the fluxes through thioredoxin-dependent reactions [3,6,11]. These results together with our modeling data (Figure 2A) suggested that changes to the thioredoxin system can affect the kinetic profiles of thioredoxin oxidation reactions. To describe this effect in more detail an analytical solution for a single cycle redoxin system with irreversible mass action kinetics for all reactions was evaluated (Scheme I). Mass action kinetic expressions were used since realistic treatments with Michaelis-Menten or ternary complex expressions for thioredoxin reductase [10] were not solvable analytically. Nonetheless these simplified solutions have showed a good correspondence to models with realistic kinetic parameters and rate expressions [10].

Scheme I

(1) NADPH+TrxSS→ NADP+TrxSH

(2) TrxSH+PSS→TrxSS+PSH

In this scheme, reaction (1) represents the reduction of oxidized thioredoxin by thioredoxin reductase (with rate *v*_1_) and reaction (2) represents the reduction of a substrate *PSS *to *PSH *with the concomitant oxidation of reduced thioredoxin to oxidized thioredoxin (with rate *v*_2_). Scheme I can be described with the following equations:

(3) $$v_{\text{1}}=k_{\text{1}}\cdot NADPH\cdot TrxSS$$

(4) $$v_{\text{2}}=k_{\text{2}}\cdot TrxSH\cdot pss$$

The analytical steady-state solution for such a single cycle redoxin system with mass action kinetics for redoxin oxidation and reduction was described previously [10]. The steady-state flux, *J*, through the thioredoxin oxidation reaction, can thus be described as follows:

(5) $$J=v_{1}=v_{2}=\frac{\left(k_{1}\cdot NADPH\cdot Trx_{total}\right)\cdot pss}{\frac{k_{1}}{k_{2}}NADPH+pss}$$

(6) $$=\frac{\left(k_{1}k_{2}\cdot NADPH\cdot Trx_{total}\right)\cdot pss}{k_{1}\cdot NADPH+k_{2}\cdot pss}$$

where *k*_1 _is rate constant for the NADPH-dependent reduction of thioredoxin, *k*_2 _is rate constant for oxidation of thioredoxin by substrate *PSS*, and *Trx_total _*is the moiety sum of the reduced and oxidized thioredoxin concentrations. Equation (5) has the same form as the Michaelis-Menten equation with an apparent *V*_max _described by *k*_1_·*NADPH*·*Trx_total_*, an apparent *k*_cat _described by *k*_1_·*NADPH *and an apparent *K*_m _described by *k*_1_·*NADPH*/*k*_2_.

As described previously [10], with increases in substrate concentration *PSS*, the flux through the thioredoxin oxidation reaction will saturate as the thioredoxin reduction reaction becomes limiting (equation (5)). Given that thioredoxin has multiple substrates within the cell [3], these results imply that a substantial increase in the concentration of these substrates, individually or collectively, could result in the apparent saturation of all substrate reduction reactions by the thioredoxin system. Secondly, the maximum flux through the thioredoxin system is determined by the concentration and activity of thioredoxin reductase (*k*_1_), the NADPH concentration, and the total thioredoxin concentration (*Trx_total_*). These molecules, together with the thioredoxin redox ratio, are therefore key indicators of the state of the thioredoxin system. The apparent *K*_m _of the thioredoxin system is determined by the relative rates of thioredoxin oxidation and reduction and the NADPH concentration (equation (5)), and if the term *k*_1_·*NADPH *is several fold higher than the term *k*_2_·*pss*, then the rate of *PSS *reduction is linear with respect to *PSS *concentration (*J *≈*k*_2_·*Trx_total_*·*pss*, see equation (6)).

**Figure 3:**
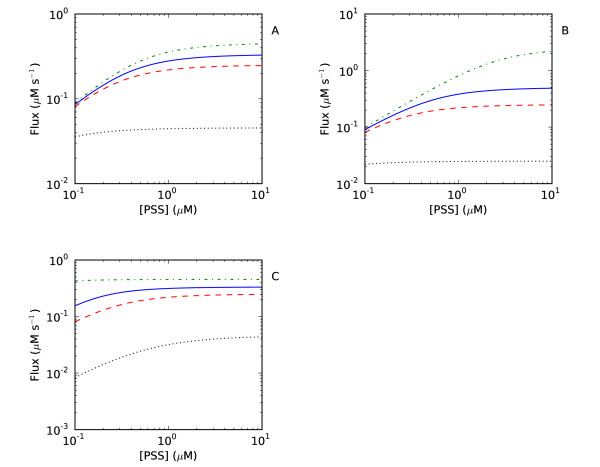
**The effect of changes in the concentration of the components of the thioredoxin system on the kinetic profile of the system**. In a kinetic model of the thioredoxin system, the concentrations of NADPH (A), thioredoxin reductase (B) and thioredoxin (C) were changed over the range 0.1 (black, •••), 1 (red, --), 2 (blue, -) and 10 (green, -•-) and the response of the flux through the thioredoxin reductase reaction towards changes in the PSS concentration monitored. Parameters were as in Table 2, except where indicated otherwise. This figure was generated using 'Logic_Figure 3.psc' (Additional file 3). A SBML version of this model ('Logic_Figure 3.xml') is available as Additional file 7.

To confirm these analytical results a core model based on Scheme I with Michaelis-Menten kinetics for the thioredoxin reductase and mass action kinetics for the thioredoxin oxidation reactions [10] was analyzed (Table 2). All parameters in the model including the total thioredoxin concentration were initially set to one. The concentration of the thioredoxin redox partner *PSS *was varied over a hundred fold range and the effect on the flux through the system determined at differing concentrations of a given parameter (Figure 3). In agreement with the analytical solution for the system (equation (5)), increasing the concentrations of NADPH and thioredoxin reductase increased the maximal attainable flux through the thioredoxin system (Figure 3A-B). Further, with increases in these concentrations, higher concentrations of *PSS *were required to saturate the system (i.e. the apparent *K*_m _for *PSS *increased (Figure 3A-B)). The activity of thioredoxin reductase is therefore a crucial determinant of the kinetic profile of thioredoxin oxidation reactions.

**Table 2:** Kinetic parameters and metabolite concentrations used in core models of the *E. coli *thioredoxin system.

	Value
**Metabolites**	(*μ*M)
NADPH	1
NADP	1
TrxSH	0.5
TrxSS	0.5
PSS	1
PSH	1
RSS	1
RSH	1
**Thioredoxin**	
**reductase**	
[TR]	1 *μ*M
*k*_cat_	1 s^-1^
*K*_nadph_	1 *μ*M
*K*_trxss_	1 *μ*M
**PSS reduction**	
*k*_2_	1 *μ*M^-1^s^-1^
**RSS reduction**	
*k*_3_	1 *μ*M^-1^s^-1^

Core models of the thioredoxin system based on Schemes I and II were created using Michaelis-Menten kinetics for thioredoxin reductase and mass action kinetics for the reduction of *PSS *and *RSS *by thioredoxin. To simplify the analysis, all reaction parameters in these models were set to one. The parameters listed in this table were used in the generation of Figures 3 and 5.

Changes in the total (oxidized and reduced) thioredoxin concentration also affected the fluxes and kinetic profiles of thioredoxin-dependent reactions in the thioredoxin system (Figure 3C). With increases in this concentration, the maximal flux through the thioredoxin system increased but the system rapidly saturated at comparatively lower concentrations of *PSS *(cf. Figure 3A-B).

### Ultrasensitivity responses in the thioredoxin system

Zero-order ultrasensitive responses are typically generated by enzymatically linked moiety-conserved cycles in which at least one of the converter enzymes is saturated by its substrate [26]. However, in our model of the *E. coli *thioredoxin system, all the reactions were coupled to a single thioredoxin redox cycle and most of the enzymatic reactions were not under zero-order conditions (Table 1). Other ultrasensitive kinetic motifs such as positive cooperativity and multi-step activation were also not evident in our kinetic model.

**Figure 4:**
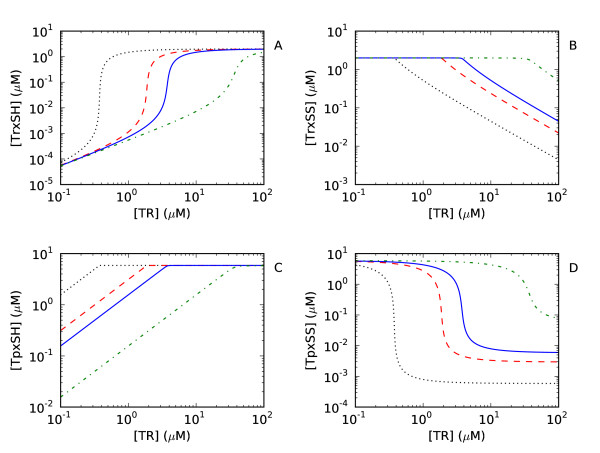
**Ultrasensitive changes in the steady state concentrations of the redox couples in the thioredoxin and Tpx redox cycles are dependent on the kinetics of the Tpx redox cycle**. In each of these simulations, the ratio of the second order rate constants for Tpx oxidation and reduction (i.e. *k*_H2O2_/*k*_trxsh_, Table 1) were varied from 0.015 (black, •••), 0.05 (red, ---), 0.10 (blue, solid line) to 1.00 (green, -•-) and the effect on the steady state concentrations of reduced thioredoxin (TrxSH), oxidized thioredoxin (TrxSS), reduced Tpx (TpxSH) and oxidized Tpx (TpxSS) monitored. Parameters were as in Table 2, except where indicated otherwise. This figure was generated using 'Logic_Figure 2_and_Figure 4.psc' (Additional file 1). A SBML version of this model ('Logic_Figure 2_and_Figure 4.xml') is available as Additional file 5.

To determine the kinetic mechanism underlying the ultrasensitive changes in the concentration of thioredoxin with changes in the thioredoxin reductase concentration (Figure 2B), the rates in each of the thioredoxin oxidation reactions in the model where were sequentially set to zero and the effect on the ultrasensitive change in the thioredoxin concentration monitored. This analysis revealed that this effect was mediated primarily by the coupling of the thioredoxin redox cycle to the Tpx redox cycle (data not shown), with the kinetic parameters of the Tpx redox cycle being critical to this response (Figure 4).

To investigate the mechanism behind this behaviour, a core model comprising just the thioredoxin and Tpx redox cycles with realistic kinetic parameters and expressions was developed. The ratio of the second order rate constants for Tpx reduction and oxidation were varied (i.e. *k*_H2O2_/*k*_trxsh_, Table 1) and the effect of the changes in the thioredoxin and Tpx redox cycles monitored in the model (Figure 4). Decreases in the *k*_H2O2_/*k*_trxsh _ratio resulted in steeper changes in the reduced thioredoxin and oxidized Tpx concentrations (Figure 4A, D) showing that the ultrasensitive response can be rationalized by considering the effects of increases in the thioredoxin reductase concentration on the Tpx moiety pool. With increases in the thioredoxin reductase concentration, the steady state rates of all the reactions in the system would be expected to increase until the thioredoxin redox cycle saturated (Figure 2A-B, see [10] for a detailed explanation). However, as the activity of hydrogen peroxide reduction reaction is far smaller than thioredoxin reduction reaction in Tpx redox cycle (i.e. *k*_H2O2_< <*k*_trxsh_, Table 1), these increases would trigger a relatively abrupt distribution of the oxidized Tpx pool into the reduced state (consider Figure 4C, k_5_/*k*_6 _= 0.010). This in turn would trigger an abrupt distribution of the thioredoxin pool into the reduced state to keep the Tpx reduction reaction at steady state, leading to an apparently ultrasensitive response. Thus, the kinetics of Tpx redox cycle determines the ultrasensitive response of the thioredoxin cycle. The hydrogen peroxide concentration used in the realistic model (Table 1) represents the basal hydrogen peroxide concentration in a wild type *E. coli *cell [27] and this ultrasensitive effect therefore occurs independently of other hydrogen peroxide scavengers such as AhpC [28].

### Interconnectivity between thioredoxin-dependent processes

In the redox circuit model of redoxin systems (Figure 1) thioredoxin-dependent reactions are considered to be essentially independent of each other. In our model of the thioredoxin system on the other hand, changes in the activity of some reactions clearly affected the fluxes of other reactions (Figure 2). This connectivity between thioredoxin-dependent pathways has been demonstrated in mammalian cells. In these cells, thioredoxin is responsible for apoptosis signal-regulating kinase 1 (Ask1) signalosome inactivation and, together with 2 Cys peroxiredoxins [16], hydrogen peroxide reduction. The oxidation of thioredoxin due to increases in the intracellular hydrogen peroxide levels triggers Ask1 signalosome activation and apoptosis [29-31] showing that these pathways do affect each other.

To describe this connectivity between thioredoxin-dependent pathways more precisely, a simplified scheme describing the oxidation of thioredoxin by two substrates and its subsequent reduction by thioredoxin reductase was analyzed:

Scheme II

(7) NADPH+TrxSS→NADP+TrxSH

(8) TrxSH+PSS→TrxSS+PSH

(9) TrxSH+RSS→TrxSS+RSH

Equations (7) and (8) were the same as in Scheme I and were described by rates *v*_1 _and *v*_2_. Reaction (8) represents an additional thioredoxin oxidation reaction, involving the reduction of *RSS *to *RSH *and was described by *v*_3_. To simplify the analysis, all reactions were described with irreversible mass action kinetics. Scheme II can be described with the following equations:

(10) $$v_{\text{1}}=k_{\text{1}}\cdot NADPH\cdot TrxSS$$

(11) $$v_{\text{2}}=k_{\text{2}}\cdot TrxSH\cdot pss$$

(12) $$v_{\text{3}}=k_{\text{3}}\cdot TrxSH\cdot rss$$

(13) $$TrxSH+TrxSS=Trx_{total}$$

(14) $$\begin{array}{l} \frac{dTrxSH}{dt}=k_{1}\;\cdot \;NADPH\;\cdot \;TrxSS\\ -k_{2}\;\cdot \;TrxSH\;\cdot \;pss-k_{3}\;\cdot \;TrxSH\;\cdot \;rss \end{array}$$

At steady state equation (14) is equal to zero and can be rearranged to yield:

(15) $$TrxSH=\frac{k_{1}\cdot NADPH\cdot Trx_{total}}{k_{1}\cdot NADPH+k_{2}\cdot pss+k_{3}\cdot rss}$$

Substituting equation (15) into equation (11) and solving yields the following the expression for the steady-state flux through reaction *v*_2_:

(16) $$J_{2}=\frac{\left(k_{1}\cdot NADPH\cdot Trx_{total}\right)\cdot pss}{\left(\frac{k_{1}\cdot NADPH+k_{3}\cdot rss}{k_{2}}\right)+pss}$$

Equation (16) is similar to the previously described analytical solution for a single cycle redoxin system (equation (5) and see [10]) with an apparent *V*_max _described by *k*_1_·*NADPH*·*Trx_total _*and an apparent *k*_cat _described by *k*_1_·*NADPH*. However, in contrast to equation (5), it includes an additional term *k_3_*·*rss*, that is part of the apparent *K*_m _for reaction *v*_2_. Using similar reasoning, it can be shown that for a system containing *n *thioredoxin oxidation reactions with their respective substrates (*XSS_i_*), the flux through *v*_2 _can be described as follows:

(17) $$J_{2}=\frac{\left(k_{1}\cdot NADPH\cdot Trx_{total}\right)\cdot pss}{\left(\frac{k_{1}\cdot NADPH+\sum_{i=3}^{n}k_{i}\cdot xss_{i}}{k_{2}}\right)+pss}$$

This result shows that the flux through reaction *v*_2 _is affected by other thioredoxin oxidation reactions. Increases in the redox partner concentrations (*XSS_i_*) of the other thioredoxin-dependent reactions can decrease the flux through a given thioredoxin oxidation reaction. In addition, it would take a higher *PSS *concentration to saturate *v*_2 _in the presence of these additional thioredoxin redox partners. In effect, these redox partners act as competitive inhibitors, increasing the apparent *K*_m _for *PSS *(cf. equation (5) and equation (17)). Finally, in a system with a single thioredoxin oxidation reaction (cf. equation (5)), the ratio of the apparent *k*_cat_/*K*_m _is equal to the second order rate constant for that thioredoxin oxidation reaction [10]. Equations (16-17) show that this relationship no longer holds for a system with more than one thioredoxin oxidation reaction, emphasizing that Michaelis-Menten parameters derived *in vitro *should be used with caution when making inferences about thioredoxin oxidation reactions *in vivo *[10], or, preferably, not used at all.

**Figure 5:**
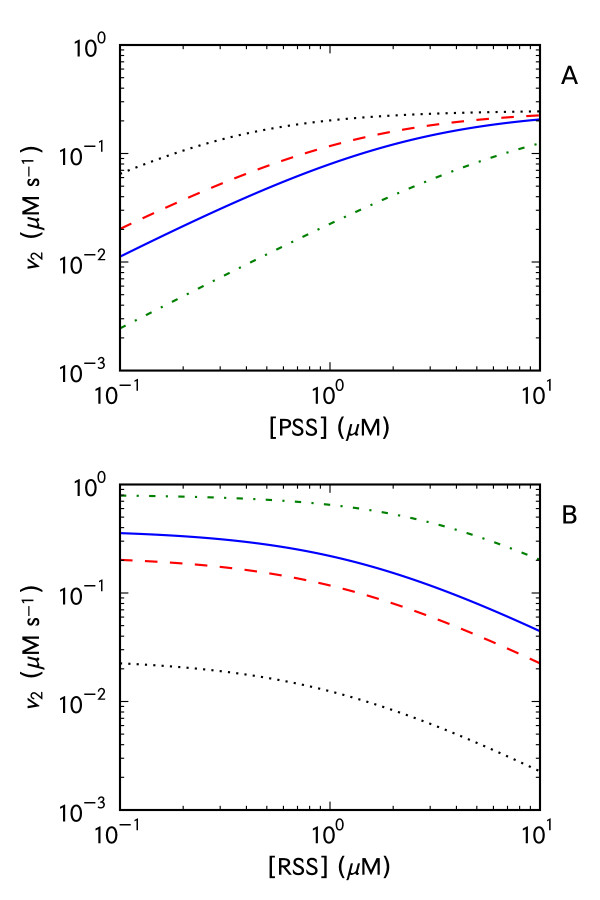
**Thioredoxin oxidation reactions can affect each other**. In (A), the flux through the thioredoxin-dependent reduction reaction of a substrate (*PSS) *at varying concentrations of a second thioredoxin substrate (*RSS*, Scheme II) was monitored in a kinetic model of the thioredoxin system (Scheme II). The concentrations of *RSS *were 0.1 (black, •••), 1 (red, --), 2 (blue, -) and 10 (green, -•-). In (B), the flux through the thioredoxin-dependent reduction of *PSS *with increasing concentrations of *RSS *was monitored over a range of thioredoxin reductase concentrations: 0.1 (black, •••), 1 (red, --), 2 (blue, -) and 10 (green, -•-). Parameters were as in Table 2, except where indicated otherwise. This figure was generated using 'Logic_Figure 5.psc' (Additional file 4). A SBML version of this model ('Logic_Figure 5.xml') is available as Additional file 8.

To confirm the analytical solution, a core model of Scheme II with Michaelis-Menten kinetics for the thioredoxin reductase and mass action kinetics for the thioredoxin oxidation reactions [10] was developed (Table 2). The effect of increasing concentrations of *RSS *on the *PSS *saturation profile for *v*_2 _was determined. As predicted by the analytical solution (equation (16)), with increases in the flux through *v*_3_, the flux through *v*_2 _decreased for a given *PSS *concentration (Figure 5A). In effect, reactions *v*_2 _and *v*_3 _were competing for the same pool of reduced thioredoxin. Further, as predicted by the analytical solution for the system, increases in the *RSS *concentration increased the apparent *K*_m _for reaction *v*_2_. Thus, thioredoxin oxidation reactions can affect the kinetic profile of other thioredoxin oxidation reactions.

Given that the thioredoxin reductase activity plays a key role in the kinetic profile of thioredoxin-dependent reactions (Figure 2A, 3B, equations (16-17)), the effect of increasing concentrations of *RSS *on the flux through reaction *v*_2 _was monitored at varying concentrations of thioredoxin reductase (Figure 5B). As described above, with increases in the concentration of *RSS*, the flux through *v*_2 _decreased but this effect was more pronounced at lower concentrations of thioredoxin reductase (Figure 5B). A high thioredoxin reductase activity ensured that reduced thioredoxin was not limiting for thioredoxin oxidation reactions and these reactions consequently exerted a smaller effect on each other.

### Redox sensitivity and insulation

The previous analyses demonstrated that all reactions linked to the thioredoxin redox cycle affect each other. However these effects were not equivalent for all reactions involving thioredoxin. In our model of the *E. coli *thioredoxin system, some reactions appeared to be readily affected by or *sensitive *to changes in the thioredoxin cycle, whilst other reactions appeared to be relatively unaffected by or *insulated *from changes to this redox cycle (Figure 2). We analyzed the effects of changes in the thioredoxin concentration on four types of reactions: protein disulfide reduction by thioredoxin, Michaelis-Menten and ping-pong enzymatic mechanisms and a thioredoxin-dependent redox cycle.

The reduction of protein disulfides by thioredoxin (Figure 1) was described with the following mass action kinetic expression:

(18) $$v=k_{1}\cdot TrxSH\cdot pss$$

This equation shows that the fluxes through thioredoxin-dependent mass action reactions change faithfully with changes in the thioredoxin concentration. Increases in the thioredoxin concentration cause a proportional increase in the flux through this reaction and *vice versa*, and such reactions are consequently very sensitive to changes in the thioredoxin redox cycle. In our *E. coli *thioredoxin kinetic model, the fluxes through the protein disulfide and PAPS reduction reactions increased or decreased with corresponding changes in the reduced thioredoxin concentration (Figure 2A-B). This reaction was however not affected by changes in the PAPS and methionine sulfoxide-reductase reactions (Figure 2D-E) as these reactions did not significantly affect the thioredoxin concentration over the concentration ranges tested (result not shown).

Thioredoxin is a substrate for enzymes such as ribonucleotide, methionine sulfoxide and PAPS-reductase [3]. The effect of changes in the thioredoxin concentration on the Michaelis-Menten and ping-pong kinetic mechanisms was analyzed. In these expressions, *a *represents the concentration of the substrate reduced with reducing equivalents from thioredoxin and *K*_a _represents the binding constant of that substrate to the enzyme. The Michaelis-Menten expression for such a reaction (assuming independent binding of the two substrates to separate sites) is shown below:

(19) $$v=\frac{V_{\max}\cdot \left(\frac{TrxSH}{K_{TrxSH}}\right)\left(\frac{a}{K_{a}}\right)}{\left(1+\frac{TrxSH}{K_{TrxSH}}\right)\left(1+\frac{a}{K_{a}}\right)}$$

The elasticity expression [21,22] of such a reaction to changes in the thioredoxin concentration is given by:

(20) $$\varepsilon_{TrxSH}^{v}=\frac{1}{1+\frac{TrxSH}{K_{TrxSH}}}$$

The ping-pong kinetic mechanism for the enzyme-catalyzed reduction of a substrate, *A*, by thioredoxin has the following expression:

(21) $$v=\frac{V_{\max}\cdot \left(\frac{TrxSH}{K_{TrxSH}}\right)\left(\frac{a}{K_{a}}\right)}{\left(\frac{a}{K_{a}}\right)+\frac{TrxSH}{K_{TrxSH}}+\left(\frac{TrxSH}{K_{TrxSH}}\right)\left(\frac{a}{K_{a}}\right)}$$

The elasticity expression of this reaction to thioredoxin is given by:

(22) $$\varepsilon_{TrxSH}^{v}=\frac{\frac{a}{K_{a}}}{\left(\frac{a}{K_{a}}+\frac{TrxSH}{K_{TrxSH}}+\frac{a\cdot TrxSH}{K_{a}K_{TrxSH}}\right)}$$

These solutions show that the sensitivity of an enzyme with Michaelis-Menten kinetics to changes in the reduced thioredoxin concentration depends on the thioredoxin concentration and binding constants (equation (20)), whilst ping-pong enzymes are sensitive to the concentrations and binding constants of both substrates (equation (22)).

Thioredoxin reduces a number of proteins such as peroxiredoxins [32,33] which are in turn oxidized by other partners, forming a redox cycle for these proteins (Figure 1). To determine the conditions under which such cycles could be insulated or sensitive to changes in the thioredoxin concentration, the following scheme was analyzed:

Scheme III

(23) $$M+TrxSH\to M^{'}+TrxSS$$

(24) $$M^{'}+C\to M+C^{'}$$

where *M *is reduced by thioredoxin and described by a mass action expression with a forward rate constant, *k*_1 _and *M' *is oxidized by *C *to *M *and the reaction described by a mass action rate expression with a forward rate constant *k*_2_. *M *and *M' *constitute a moiety conserved cycle with moiety sum, *M*_t_. As described for equation (5), the steady-state flux through the the reduction of *C *by *M' *can be described by the following kinetic expression:

(25) $$J=\frac{k_{1}\cdot TrxSH\cdot M_{\text{t}}\cdot c}{\frac{k_{1}}{k_{2}}TrxSH+c}$$

The response coefficient [34] for this expression with respect to thioredoxin is

(26) $$R_{TrxSH}^{J}=\frac{c}{\frac{k_{1}}{k_{2}}TrxSH+c}$$

This analysis indicates that if the concentration of the oxidizing substrate for the redox cycle (*c*, Scheme III) is very low, then equation (26) tends to zero and the cycle is relatively insensitive to changes in the reduced thioredoxin concentration. On the other hand, if the concentration of the oxidizing substrate was to increase (relative to the term *k*_1_/*k*_2_*TrxSH*), then the response would approach one indicating that the cycle would be more sensitive to the reduced thioredoxin concentration and therefore the thioredoxin redox cycle. Equation (26) also shows that if the ratio of the reduction and oxidation rate constants for *M *and *M' *respectively (i.e. *k*_1_/*k*_2_, equation (26)) is very high compared to the concentration of *c*, then the flux through the redox cycle is also relatively insulated from changes in the concentration of reduced thioredoxin. Thus, those cycles with a higher rate of thioredoxin-dependent reduction (cf. equation (23)) compared to oxidation (cf. equation (24)) are less sensitive to changes in the thioredoxin redox cycle. Finally and in agreement with other modeling results (Figure 5B), this equation shows that limiting concentrations of (reduced) thioredoxin increase the response of this cycle to changes in the thioredoxin redox cycle.

In our computational model of the *E. coli *thioredoxin system (Figure 2), the rate constant for Tpx reduction (3000 *μ*M.s^-1^) was significantly greater than the rate constant for Tpx oxidation (44 *μ*M.s^-1^) (Table 1). As predicted from equation (26), this redox cycle was consequently less sensitive to changes in the thioredoxin redox cycle compared to other thioredoxin oxidation reactions (Figure 2). Further, the comparatively high rate of thioredoxin oxidation by Tpx also ensured that in the presence of hydrogen peroxide, reducing equivalents from thioredoxin were preferentially used for hydrogen peroxide reduction and that the fluxes of other thioredoxin oxidation reactions were decreased (Figure 2F).

## Discussion

The thioredoxin system appears to be regulated at multiple levels from gene expression [11,35,36] to the cellular metabolism [7,37] level. This, together with large number of thioredoxin-dependent processes [3], suggests a potentially complex network of regulatory interactions. In this paper, we focused on the kinetic regulation of the *E. coli *thioredoxin system and associated reactions.

Changes in the component concentrations of the thioredoxin system *in vitro *have resulted in changes to the Michaelis-Menten parameters assigned to thioredoxin [8], whilst changes to concentrations and activities of components of the thioredoxin system *in vivo *have been associated with distinct physiological changes [3,8,38-42]. These results suggest that the thioredoxin system is adaptable and our analysis confirmed that changes in the system, especially the thioredoxin reductase concentration, can affect the kinetic profiles of thioredoxin oxidation reactions. For example, decreases in the thioredoxin reductase activity decreased the fluxes and caused thioredoxin oxidation reactions to saturate at lower concentrations of their substrates (Figures 2A, 3B). In addition, the fluxes of thioredoxin oxidation reactions affected each other to a greater extent (Figure 5B). Thioredoxin reductase activity is therefore a key parameter for the function of the thioredoxin system and associated reactions. These results explain why drugs that target this enzyme are so potent [41,42] as inhibition of thioredoxin reductase would not only affect the thioredoxin redox cycle but could trigger system-wide effects on all thioredoxin-dependent reactions (Figure 2A). The viability *E. coli *and yeast thioredoxin reductase mutants [3,43] does however suggest that the glutathione/glutaredoxin system can adequately compensate for the thioredoxin system in these cells [3].

Despite evidence of cross-talk between thioredoxin pathways in Ask1 signalosome activation [29-31], thioredoxin-dependent reactions and processes have been considered essentially independent of each other in redox circuit models of the thioredoxin system (see for example Figures 1, 2 in [3] and Figure 1 in [42]). However, our analysis showed that changes in the thioredoxin cycle which increased or decreased the steady state thioredoxin concentration, affected the fluxes of other thioredoxin oxidation reactions (Figure 2, equations (16-17)). Thioredoxin oxidation reactions are therefore connected to, and can affect each other via the thioredoxin redox cycle. Whilst all thioredoxin-dependent reactions can, in principle, affect each other, our model of the *E. coli *thioredoxin system showed that these reactions displayed differential sensitivities to changes in the redox cycle, allowing for several modes of kinetic regulation within the system (Figure 2). Thioredoxin-dependent mass action reactions were relatively sensitive to changes in the thioredoxin redox cycle (Figure 2A, F), whilst the sensitivity of enzymatic reactions and redox cycles (Scheme II) depended on their individual kinetic parameters (equations (20, 22, 26), Figure 2A, C, E, F). For example, in response to increasing hydrogen peroxide concentrations, non-specific protein disulfide reduction by thioredoxin was dramatically reduced, whilst the Tpx flux, which is involved in the oxidative stress response, was affected to a lesser degree (Figure 2F). These results suggest that the flux distribution within the thioredoxin system may change dynamically in response to the physiological state of the cell.

In our kinetic model the Tpx redox cycle in particular was insulated from changes that occurred in the thioredoxin redox cycle when compared to other thioredoxin oxidation reactions (Figure 2). Further, the relatively high rate constant for the thioredoxin-dependent reduction of Tpx (Table 1) ensured that hydrogen peroxide reduction was prioritized over protein disulfide, PAPS and methionine sulfoxide reduction (Figure 2F). This finding agrees with other work showing that *E. coli *metabolic processes are very sensitive to hydrogen peroxide (reviewed in [44]) and the model predicts that the coupling of hydrogen peroxide metabolism to the thioredoxin redox cycle can limit sensitive cellular processes, such as DNA synthesis, under oxidative stress conditions.

An intriguing finding was that the kinetic structure of the Tpx redox cycle and its coupling to the thioredoxin redox cycle, led to ultrasensitive changes in the reduced thioredoxin concentration with changes in the thioredoxin reductase concentration. In turn, these changes resulted in large changes in the fluxes of thioredoxin-dependent cellular processes (Figures 2A-B). This indicates that the coupling between hydrogen peroxide metabolism and the thioredoxin redox cycle could represent a mechanism to coordinate large changes in the thioredoxin-dependent processes within a cell and that the inhibition of Tpx activity would disrupt this mechanism. Whether this mechanism is utilized *in vivo *still needs to be determined although the upregulation of thioredoxin reductase in pathological conditions such as cancer [2] suggests that this may represent a mechanism for these cells to effect large changes in their metabolism.

## Conclusions

In keeping with published findings, our analysis shows that the kinetics of the thioredoxin redox cycle allows for a system that is adaptable and, in contrast to other models of the system, connects thioredoxin-dependent processes. This is significant because it shows that the capacity through thioredoxin-dependent pathways depends critically on the concentration of thioredoxin reductase and that the electron circuit approach to modeling the thioredoxin system (Figure 1) is limited. Depending on their kinetics thioredoxin-dependent reactions can show differential sensitivities to changes in the thioredoxin redox cycle, allowing for several modes of kinetic regulation within the system. Taken together, these results indicate that the thioredoxin redox cycle is an analogue device that receives, distributes and coordinates redox signaling between metabolic processes within the cell. This work serves as a good starting point for the experimental analysis of the network properties of the thioredoxin system.

## Methods

### Kinetic Modeling

All kinetic modeling experiments were carried out using the open source Python Simulator for Cellular Systems (PySCeS) modeling software [45]. Three types of models were used for analyzing the thioredoxin system. Models with mass action kinetics for all reactions were used to derive analytical solutions for the thioredoxin system. These solutions were confirmed with core models that contained realistic kinetic expressions for thioredoxin reductase but were parameterized with default parameter sets (i.e. all parameters were set to one). Finally, a realistic kinetic parameter set was used to create a computational model of the *E. coli *thioredoxin system (described below). To describe the kinetic behavior of the thioredoxin system at the pathway level, core and realistic model outputs were plotted as parameter portraits, which capture the systems-level behavior of a metabolic pathway (for a detailed description and examples see [21,46]). All models will be made available on the JWS Online database [47] and are available in PySCeS (Additional files 1, 2, 3 and 4) and SBML (Additional files 5, 6, 7 and 8) formats. PySCeS model outputs were confirmed with Copasi [48]. In all reaction schemes species names were capitalized, whilst lower case was used to describe species concentrations.

### Realistic kinetic model of the *E. coli *thioredoxin system

A realistic kinetic model of the *E. coli *thioredoxin system was developed that included a number of thioredoxin-dependent reactions: thioredoxin reductase, methionine sulfoxide reductase, PAPS reductase, Tpx and protein disulfide reduction (Figure 1). The kinetic parameters for the model were obtained from literature and from the BRENDA [49] and CyberCell databases [50] (Table 1). However, the concentration of oxidized disulfides (PSS), methionine sulfoxide (MetSO), methionine sulfoxide reductase ([MsrA]), PAPS and PAPS reductase ([PR]) could not be obtained directly from the literature and were estimated as follows.

To estimate the concentration of oxidized disulfides we used a dataset that identified the redox partners of the thioredoxin system in *E. coli *[51]. Protein sequences for these redox partners were obtained from Uniprot http://www.uniprot.org/ and the numbers of possible disulfide bridges in these proteins were estimated using the DiANNA web server http://clavius.bc.edu/~clotelab/DiANNA/[52,53]. The *in vivo *concentration of these disulfide bridges were then estimated using protein concentrations from the CyberCell database [50] which gave a disulfide concentration of 423 *μ*M. This value represents complete oxidation of all the disulfides in these redox partners and for the modeling experiments shown here we assumed that 1% of the target disulfides were oxidized. Simulations of the model were undertaken with higher concentration of oxidized disulfide and the results obtained were in agreement the main findings described above (results not shown).

Under normal cultivation conditions 2% of methionine in the *E. coli *proteome is oxidized to methionine sulfoxide [54], although the actual concentration of this methionine sulfoxide is not known. Assuming a total *E. coli *protein concentration of 250 mg/ml [55] and an average methionine content in the proteome of 2.9% [56], we estimated a proteome methionine concentration of 48.3 mM and a methionine sulfoxide concentration of 0.97 mM. The intracellular protein concentration of methionine sulfoxide reductase was estimated by taking the ratio of its copy number to the Tpx copy number and multiplying by the intracellular concentration of Tpx [57]. The concentration of Tpx was varied ten-fold during simulations of the model but this did not affect the main findings of the study (results not shown).

PAPS reductase is encoded by *cysH *which is part of the *cysCDHIJ *regulon [58] and its concentration was estimated by taking the ratio of its copy number to the CysI copy number and multiplying by the intracellular concentration of CysI [50]. The concentration of PAPS within *E. coli *was not known but has been shown to vary from 0.07 to 20 *μ*M in *Saccharomyces cerevisiae *depending on the state of the cell [59,60]. We used 0.07 *μ*M in this study. While PAPS reductase has a ping-pong kinetic mechanism [12] it was modeled with mass action kinetics (Figures 2A-C, E, F) because our assigned concentration was almost 5-fold higher than the concentration of its substrate (Table 1). However, to determine the effect of increasing PAPS concentrations on the thiroedoxin system, the PAPS reductase reaction was modeled with ping-pong kinetics (Figure 2D).

## Authors' contributions

CSP conceived the study, performed the kinetic modeling and drafted the manuscript, J-HSH and JMR participated in the kinetic analysis and drafted the manuscript. All authors read and approved the final manuscript.

## References

[B1] Vlamis-Gardikas A The multiple functions of the thiol-based electron flow pathways of Escherichia coli: Eternal concepts revisited Biochim Biophys Acta 2008 1780 1170 1200 18423382 10.1016/j.bbagen.2008.03.013

[B2] Arner ES Holmgren A The thioredoxin system in cancer Semin Cancer Biol 2006 16 420 426 10.1016/j.semcancer.2006.10.009 17092741

[B3] Toledano MB Kumar C Le Moan N Spector D Tacnet F The system biology of thiol redox system in Escherichia coli and yeast: differential functions in oxidative stress, iron metabolism and DNA synthesis FEBS Lett 2007 581 3598 3607 10.1016/j.febslet.2007.07.002 17659286

[B4] Holmgren A Bjornstedt M Thioredoxin and thioredoxin reductase Methods Enzymol 1995 252 199 208 full_text 7476354 10.1016/0076-6879(95)52023-6

[B5] Arner ES Holmgren A Physiological functions of thioredoxin and thioredoxin reductase Eur J Biochem 2000 267 6102 6109 10.1046/j.1432-1327.2000.01701.x 11012661

[B6] Trotter EW Grant CM Non-reciprocal regulation of the redox state of the glutathione-glutaredoxin and thioredoxin systems EMBO Rep 2003 4 184 188 10.1038/sj.embor.embor729 12612609 PMC1315827

[B7] Kemp M Go YM Jones DP Nonequilibrium thermodynamics of thiol/disulfide redox systems: a perspective on redox systems biology Free Radic Biol Med 2008 44 921 937 10.1016/j.freeradbiomed.2007.11.008 18155672 PMC2587159

[B8] Holmgren A Reduction of disulfides by thioredoxin. Exceptional reactivity of insulin and suggested functions of thioredoxin in mechanism of hormone action J Biol Chem 1979 254 9113 9119 39074

[B9] Zahedi Avval F Holmgren A Molecular mechanisms of thioredoxin and glutaredoxin as hydrogen donors for mammalian S-phase ribonucleotide reductase J Biol Chem 2009 284 8233 8240 10.1074/jbc.M809338200 19176520 PMC2659180

[B10] Pillay CS Hofmeyr JH Olivier BG Snoep JL Rohwer JM Enzymes or redox couples? The kinetics of thioredoxin and glutaredoxin reactions in a systems biology context Biochem J 2009 417 269 275 10.1042/BJ20080690 18694397

[B11] Potamitou A Holmgren A Vlamis-Gardikas A Protein levels of Escherichia coli thioredoxins and glutaredoxins and their relation to null mutants, growth phase, and function J Biol Chem 2002 277 18561 18567 10.1074/jbc.M201225200 11893749

[B12] Boschi-Muller S Azza S Branlant G E. coli methionine sulfoxide reductase with a truncated N terminus or C terminus, or both, retains the ability to reduce methionine sulfoxide Protein Sci 2001 10 2272 2279 10.1110/ps.10701 11604533 PMC2374066

[B13] Boschi-Muller S Olry A Antoine M Branlant G The enzymology and biochemistry of methionine sulfoxide reductases Biochim Biophys Acta 2005 1703 231 238 15680231 10.1016/j.bbapap.2004.09.016

[B14] Lillig CH Prior A Schwenn JD Aslund F Ritz D Vlamis-Gardikas A Holmgren A New thioredoxins and glutaredoxins as electron donors of 3'- phosphoadenylylsulfate reductase J Biol Chem 1999 274 7695 7698 10.1074/jbc.274.12.7695 10075658

[B15] Baker LM Poole LB Catalytic mechanism of thiol peroxidase from Escherichia coli. Sulfenic acid formation and overoxidation of essential CYS61 J Biol Chem 2003 278 9203 9211 10.1074/jbc.M209888200 12514184 PMC3845838

[B16] Winterbourn CC Hampton MB Thiol chemistry and specificity in redox signaling Free Radic Biol Med 2008 45 549 561 10.1016/j.freeradbiomed.2008.05.004 18544350

[B17] Ogusucu R Rettori D Munhoz DC Netto LE Augusto O Reactions of yeast thioredoxin peroxidases I and II with hydrogen peroxide and peroxynitrite: rate constants by competitive kinetics Free Radic Biol Med 2007 42 326 334 10.1016/j.freeradbiomed.2006.10.042 17210445

[B18] Parsonage D Karplus PA Poole LB Substrate specificity and redox potential of AhpC, a bacterial peroxiredoxin Proc Natl Acad Sci USA 2008 105 8209 8214 10.1073/pnas.0708308105 18165315 PMC2448816

[B19] Sayed AA Williams DL Biochemical characterization of 2-Cys peroxiredoxins from Schistosoma mansoni J Biol Chem 2004 279 26159 26166 10.1074/jbc.M401748200 15075328

[B20] Johnson RM Goyette G Jr Ravindranath Y Ho YS Hemoglobin autoxidation and regulation of endogenous H2O2 levels in erythrocytes Free Radic Biol Med 2005 39 1407 1417 10.1016/j.freeradbiomed.2005.07.002 16274876

[B21] Hofmeyr JS Cornish-Bowden A Regulating the cellular economy of supply and demand FEBS Lett 2000 476 47 51 10.1016/S0014-5793(00)01668-9 10878248

[B22] Hofmeyr JH Metabolic regulation: a control analytic perspective J Bioenerg Biomembr 1995 27 479 490 10.1007/BF02110188 8718453

[B23] Bersani NA Merwin JR Lopez NI Pearson GD Merrill GF Protein electrophoretic mobility shift assay to monitor redox state of thioredoxin in cells Methods Enzymol 2002 347 317 326 full_text 11898422 10.1016/s0076-6879(02)47031-0

[B24] Derman AI Beckwith J Escherichia coli alkaline phosphatase localized to the cytoplasm slowly acquires enzymatic activity in cells whose growth has been suspended: a caution for gene fusion studies J Bacteriol 1995 177 3764 3770 7601842 10.1128/jb.177.13.3764-3770.1995 PMC177094

[B25] Stewart EJ Aslund F Beckwith J Disulfide bond formation in the Escherichia coli cytoplasm: an in vivo role reversal for the thioredoxins EMBO J 1998 17 5543 5550 10.1093/emboj/17.19.5543 9755155 PMC1170883

[B26] Goldbeter A Koshland DE Jr Ultrasensitivity in biochemical systems controlled by covalent modification. Interplay between zero-order and multistep effects J Biol Chem 1984 259 14441 14447 6501300

[B27] Seaver LC Imlay JA Hydrogen peroxide fluxes and compartmentalization inside growing Escherichia coli J Bacteriol 2001 183 7182 7189 10.1128/JB.183.24.7182-7189.2001 11717277 PMC95567

[B28] Seaver LC Imlay JA Alkyl hydroperoxide reductase is the primary scavenger of endogenous hydrogen peroxide in Escherichia coli J Bacteriol 2001 183 7173 7181 10.1128/JB.183.24.7173-7181.2001 11717276 PMC95566

[B29] Nadeau PJ Charette SJ Toledano MB Landry J Disulfide Bond-mediated multimerization of Ask1 and its reduction by thioredoxin-1 regulate H(2)O(2)-induced c-Jun NH(2)-terminal kinase activation and apoptosis Mol Biol Cell 2007 18 3903 3913 10.1091/mbc.E07-05-0491 17652454 PMC1995733

[B30] Liu H Zhang H Iles KE Rinna A Merrill G Yodoi J Torres M Forman HJ The ADP-stimulated NADPH oxidase activates the ASK-1/MKK4/JNK pathway in alveolar macrophages Free Radic Res 2006 40 865 874 10.1080/10715760600758514 17015265 PMC2713795

[B31] Fujino G Noguchi T Takeda K Ichijo H Thioredoxin and protein kinases in redox signaling Semin Cancer Biol 2006 16 427 435 10.1016/j.semcancer.2006.09.003 17081769

[B32] Janssen-Heininger YM Mossman BT Heintz NH Forman HJ Kalyanaraman B Finkel T Stamler JS Rhee SG van der Vliet A Redox-based regulation of signal transduction: principles, pitfalls, and promises Free Radic Biol Med 2008 45 1 17 10.1016/j.freeradbiomed.2008.03.011 18423411 PMC2453533

[B33] Rhee SG Chae HZ Kim K Peroxiredoxins: a historical overview and speculative preview of novel mechanisms and emerging concepts in cell signaling Free Radic Biol Med 2005 38 1543 1552 10.1016/j.freeradbiomed.2005.02.026 15917183

[B34] Fell D Understanding the control of metabolism 1997 London: Portland Press

[B35] Miranda-Vizuete A Rodriguez-Ariza A Toribio F Holmgren A Lopez-Barea J Pueyo C The levels of ribonucleotide reductase, thioredoxin, glutaredoxin 1, and GSH are balanced in Escherichia coli K12 J Biol Chem 1996 271 19099 19103 10.1074/jbc.271.32.19099 8702583

[B36] Prieto-Alamo MJ Jurado J Gallardo-Madueno R Monje-Casas F Holmgren A Pueyo C Transcriptional regulation of glutaredoxin and thioredoxin pathways and related enzymes in response to oxidative stress J Biol Chem 2000 275 13398 13405 10.1074/jbc.275.18.13398 10788450

[B37] Jones DP Radical-free biology of oxidative stress Am J Physiol Cell Physiol 2008 295 C849 868 10.1152/ajpcell.00283.2008 18684987 PMC2575825

[B38] Kaimul AM Nakamura H Masutani H Yodoi J Thioredoxin and thioredoxin-binding protein-2 in cancer and metabolic syndrome Free Radic Biol Med 2007 43 861 868 10.1016/j.freeradbiomed.2007.05.032 17697931

[B39] Singh K Kang PJ Park HO The Rho5 GTPase is necessary for oxidantinduced cell death in budding yeast Proc Natl Acad Sci USA 2008 105 1522 1527 10.1073/pnas.0707359105 18216266 PMC2234177

[B40] Cox AG Brown KK Arner ES Hampton MB The thioredoxin reductase inhibitor auranofin triggers apoptosis through a Bax/Bak-dependent process that involves peroxiredoxin 3 oxidation Biochem Pharmacol 2008 76 1097 1109 10.1016/j.bcp.2008.08.021 18789312

[B41] Lu J Chew EH Holmgren A Targeting thioredoxin reductase is a basis for cancer therapy by arsenic trioxide Proc Natl Acad Sci USA 2007 104 12288 12293 10.1073/pnas.0701549104 17640917 PMC1940330

[B42] Powis G Kirkpatrick DL Thioredoxin signaling as a target for cancer therapy Curr Opin Pharmacol 2007 7 392 397 10.1016/j.coph.2007.04.003 17611157

[B43] Fuchs J Isolation of an Escherichia coli mutant deficient in thioredoxin reductase J Bacteriol 1977 129 967 972 14115 10.1128/jb.129.2.967-972.1977 PMC235035

[B44] Imlay JA Cellular defenses against superoxide and hydrogen peroxide Annu Rev Biochem 2008 77 755 776 10.1146/annurev.biochem.77.061606.161055 18173371 PMC3057177

[B45] Olivier BG Rohwer JM Hofmeyr JH Modelling cellular systems with PySCeS Bioinformatics 2005 21 560 561 10.1093/bioinformatics/bti046 15454409

[B46] du Preez FB Conradie R Penkler GP Holm K van Dooren FL Snoep JL A comparative analysis of kinetic models of erythrocyte glycolysis J Theor Biol 2008 252 488 496 10.1016/j.jtbi.2007.10.006 18031761

[B47] Olivier BG Snoep JL Web-based kinetic modelling using JWS Online Bioinformatics 2004 20 2143 2144 10.1093/bioinformatics/bth200 15072998

[B48] Hoops S Sahle S Gauges R Lee C Pahle J Simus N Singhal M Xu L Mendes P Kummer U COPASI--a COmplex PAthway SImulator Bioinformatics 2006 22 3067 3074 10.1093/bioinformatics/btl485 17032683

[B49] Chang A Scheer M Grote A Schomburg I Schomburg D BRENDA, AMENDA and FRENDA the enzyme information system: new content and tools in 2009 Nucleic Acids Res 2009 37 D588 592 10.1093/nar/gkn820 18984617 PMC2686525

[B50] Sundararaj S Guo A Habibi-Nazhad B Rouani M Stothard P Ellison M Wishart DS The CyberCell Database (CCDB): a comprehensive, selfupdating, relational database to coordinate and facilitate in silico modeling of Escherichia coli Nucleic Acids Res 2004 32 D293 295 10.1093/nar/gkh108 14681416 PMC308842

[B51] Leichert LI Jakob U Protein thiol modifications visualized in vivo PLoS Biol 2004 2 e333 10.1371/journal.pbio.0020333 15502869 PMC521172

[B52] Ferre F Clote P DiANNA 1.1: an extension of the DiANNA web server for ternary cysteine classification Nucleic Acids Res 2006 34 W182 185 10.1093/nar/gkl189 16844987 PMC1538812

[B53] Ferre F Clote P DiANNA: a web server for disulfide connectivity prediction Nucleic Acids Res 2005 33 W230 232 10.1093/nar/gki412 15980459 PMC1160173

[B54] Rosen H Klebanoff SJ Wang Y Brot N Heinecke JW Fu X Methionine oxidation contributes to bacterial killing by the myeloperoxidase system of neutrophils Proc Natl Acad Sci USA 2009 106 18686 18691 10.1073/pnas.0909464106 19833874 PMC2774013

[B55] Zimmerman SB Trach SO Estimation of macromolecule concentrations and excluded volume effects for the cytoplasm of Escherichia coli J Mol Biol 1991 222 599 620 10.1016/0022-2836(91)90499-V 1748995

[B56] Neidhardt FC Umbarger HE Eds Chemical composition of Escherichia coli 1996 2 Washington, D.C.: ASM Press

[B57] Link AJ Robison K Church GM Comparing the predicted and observed properties of proteins encoded in the genome of Escherichia coli K-12 Electrophoresis 1997 18 1259 1313 10.1002/elps.1150180807 9298646

[B58] Tei H Murata K Kimura A Molecular cloning of the cys genes (cysC, cysD, cysH, cysI, cysJ, and cysG) responsible for cysteine biosynthesis in Escherichia coli K-12 Biotechnol Appl Biochem 1990 12 212 216 2158792

[B59] Murguia JR Belles JM Serrano R The yeast HAL2 nucleotidase is an in vivo target of salt toxicity J Biol Chem 1996 271 29029 29033 10.1074/jbc.271.46.29029 8910555

[B60] Jakubowski H Goldman E Methionine-mediated lethality in yeast cells at elevated temperature J Bacteriol 1993 175 5469 5476 8366032 10.1128/jb.175.17.5469-5476.1993 PMC206603

[B61] Ishii N Nakahigashi K Baba T Robert M Soga T Kanai A Hirasawa T Naba M Hirai K Hoque A Ho PY Kakazu Y Sugawara K Igarashi S Harada S Masuda T Sugiyama N Togashi T Hasegawa M Takai Y Yugi K Arakawa K Iwata N Toya Y Nakayama Y Nishioka T Shimizu K Mori H Tomita M Multiple high-throughput analyses monitor the response of E. coli to perturbations Science 2007 316 593 597 10.1126/science.1132067 17379776

[B62] Chinn PC Pigiet V Fahey RC Determination of thiol proteins using monobromobimane labeling and high-performance liquid chromatographic analysis: application to Escherichia coli thioredoxin Anal Biochem 1986 159 143 149 10.1016/0003-2697(86)90319-2 3544950

[B63] Gleason FK Lim CJ Gerami-Nejad M Fuchs JA Characterization of Escherichia coli thioredoxins with altered active site residues Biochemistry 1990 29 3701 3709 10.1021/bi00467a016 2187529

[B64] Williams CHJ Flavin-Containing Dehydrogenases 1976 New York: Academic Press

